# A novel anti-cancer effect of resveratrol: reversal of epithelial-mesenchymal transition in prostate cancer cells

**DOI:** 10.3892/mmr.2014.2417

**Published:** 2014-07-23

**Authors:** JIANPING LI, TIE CHONG, ZIMING WANG, HAIWEN CHEN, HECHENG LI, JUN CAO, PENG ZHANG, HONGLIANG LI

**Affiliations:** Department of Urology, The Second Affiliated Hospital of Medical College, Xi’an Jiaotong University, Xi’an, Shaanxi 710004, P.R. China

**Keywords:** resveratrol, prostate cancer, invasion, epithelial-mesenchymal transition

## Abstract

Carcinoma progression is associated with the loss of epithelial features and the acquisition of a mesenchymal phenotype, a process known as epithelial-mesenchymal transition (EMT). Resveratrol, a natural polyphenolic compound found in grapes, berries and peanuts, has a wide range of pharmacological properties, including anti-tumor metastasis properties. The underlying mechanism through which resveratrol inhibits metastasis of prostate cancer (PCa) is not yet fully understood; however, it is thought to be associated with the disruption of EMT. In the present study, lipopolysaccharide (LPS) was used to trigger EMT in PC-3 and LNCaP PCa cell lines, and the cell lines were subsequently treated with resveratrol. The results demonstrated that exposure of PC-3 and LNCaP cells to LPS resulted in morphological alterations characteristic of EMT, as well as an increase in the expression of the mesenchymal marker vimentin and a decrease in the expression of E-cadherin. In addition, LPS exposure resulted in an increase in cell motility, along with an upregulation of the transcription factor glioma-associated oncogene homolog 1 (Gli1). However, treatment with resveratrol inhibited LPS-induced morphological changes, decreased the expression of LPS-induced markers of EMT and inhibited the expression of Gli1, resulting in the inhibition of *in vitro* cell motility and invasiveness. These results provide a novel perspective for the anti-invasion mechanism of resveratrol, suggesting that the effect is in part due to its ability to inhibit the EMT process through the Hedgehog signaling pathway.

## Introduction

Prostate cancer (PCa) is one of the most common malignancies and represents the second most common cause of cancer-associated mortalities among males in the USA (1). Metastasis is a major cause of mortality among patients with PCa (2). Clinically insignificant tumors are widespread in elderly men; however, PCa frequently has an aggressive phenotype that requires rapid intervention (3). Therefore, PCa is a target for effective anti-metastatic drugs.

Epithelial-mesenchymal transition (EMT) is a fundamental process of embryogenesis; however, it is also associated with the progression of a number of different types of cancer (4–6). During EMT reprogramming, epithelial cells acquire mesenchymal phenotypes. In addition, they gain the expression of mesenchymal markers, including vimentin, fibronectin and N-cadherin, which results in an enhanced ability for cell migration and invasion (7), therefore promoting the metastatic ability of cancer cells. Following migration, the tumor cells undergo mesenchymal-to-epithelial transition (MET) and regain the expression of epithelial markers, including E-cadherin (8). Increasing evidence suggests that EMT is important during the progression and malignant transformation of PCa, allowing cancer cells to gain invasive and metastatic properties (9–11). Therefore, EMT may be a promising therapeutic target and the inhibition of EMT may prevent or restrain the invasion and metastasis of PCa cells.

Current anti-cancer therapies only offer modest benefits (12) and natural products as chemopreventive agents may provide alternative and safe cancer treatments (13). Among several dietary chemopreventive agents, resveratrol has gained considerable interest (14). Resveratrol is found in various plants, including grapes, and is used in traditional Chinese medicine (15). Previous studies have shown that resveratrol has numerous pharmaceutical properties, including anti-tumorigenic capabilities (16) against a number of different tumor cell types, including breast, prostate and esophageal cancer cells (17–19). Resveratrol is also considered to be a potent chemopreventive agent. It has been suggested that the anti-invasive effects of resveratrol may be due to its ability to inhibit EMT; however, the molecular mechanisms of resveratrol have yet to be fully elucidated. It has previously been shown that lipopolysaccharide (LPS) induces EMT in cancer cells (20), therefore, in the present study, LPS was used to induce EMT. The aim of the present study was to investigate the ability of resveratrol to inhibit LPS-triggered EMT in PCa cells. In addition, it was investigated whether this effect was accompanied by the inhibition of the Hedgehog (Hh) signaling pathway.

## Materials and methods

### Reagents and cell culture

Antibodies against glioma-associated oncogene homolog 1 (Gli1), E-cadherin, vimentin and β-actin were purchased from Santa Cruz Biotechnology, Inc. (Santa Cruz, CA, USA). Matrigel and LPS were purchased from Sigma (St. Louis, MO, USA). Resveratrol (purity, >98%) was purchased from Shanghai Tauto Biotech Co., Ltd. (Shanghai, China). PC-3 and LNCaP prostate cancer cell lines (obtained from the American Type Culture Collection, Manassas, VA, USA) were maintained in Dulbecco’s modified Eagle medium (DMEM, Gibco^®^, Carlsbad, CA, USA) supplemented with penicillin (100 U/ml), streptomycin (100 μg/ml), 0.1 mM non-essential amino acids, 0.2 mM glutamine, 1 mM pyruvate and 10% heat-inactivated fetal bovine serum and incubated in 5% CO_2_ humidified atmosphere at 37°C. Cells were grown to 80% confluence prior to treatment. The present study was approved by the Institutional Review Board and Ethics Committee of the Xi’an Jiaotong University, Xi’an, China.

### Cell viability assay

Cells were seeded (5×10^3^/well) in 200 μl of DMEM into 96-well plates and cultured overnight. The MTT assay was then used to determine cell viability. Resveratrol (0–50 μM) was added to the cells and the cells were cultured for 24 h. The MTT reagent (5 mg/ml) was added and the cells were incubated for a further 4 h. The reaction was terminated by adding 150 μl dimethylsulfoxide (Sigma) per well. The absorbance values were determined using an MRX Revelation 96-well multiscanner (Dynex Technologies, Chantilly, VA, USA). The cells cultured in DMEM served as the control group. The cell viability index was calculated using the following formula: Experimental optical density (OD) value/control OD value. Each experiment was repeated three times.

### Scanning electron microscopy

The cells treated or untreated with resveratrol were harvested and washed with phosphate-buffered saline (PBS). Cells were fixed for 2 h in 4% paraformaldehyde and 1% glutaraldehyde in 0.1 M phosphate buffer (PB; pH 7.4) and then rinsed in PB, prior to being fixed in 1% osmium tetraoxide for 1 h. The cells were washed with PB and progressively dehydrated in 10% graded series of 30–100% ethanol. The cells were then dried in an acetonitrile solution of 70–100%. Finally, cells were sprayed gold and examined using scanning electron microscopy.

### Immunofluorescence assay

Exponentially growing cells were seeded on 25-mm square glass cover slips and placed in 35-mm diameter culture dishes. Following treatment, the cells were fixed with 4% formaldehyde for 5 min, permeabilized with 0.2% solution of Triton X-100 in PBS and blocked using 2% bovine serum albumin-PBS for 30 min. Slides were then incubated overnight with cyanine 3-labeled anti-E-cadherin (1:100) and fluorescein isothiocyanate-labeled anti-vimentin (1:100), respectively. The cell nuclei were counterstained using DAPI. Fluorescent imaging was performed using a confocal laser scanning microscope (Carl Zeiss MicroImaging, Inc., Oberkochen, Germany).

### Cell invasion assay

The cell invasion assay was performed using Boyden chambers with 8 μm porosity polyvinylpyrrolidone-free polycarbonate filters coated with 50 μg/ml Matrigel solution. The cells were seeded in 12-well plates at a concentration of 2.5×10^5^/well and were cultured for 48 h with LPS (5 μg/ml). For the co-treatment experiment, 20 μM resveratrol was added to the cell cultures 1 h prior to the addition of LPS. Normal culture medium was added to the bottom chamber to induce the cancer cell lines. Pretreated cells were seeded in the top chamber. The Matrigel invasion chamber was incubated for 24 h in a humidified tissue culture incubator, and after 24 h, the non-invasive cells were removed from the upper surface of the separating membrane using a cotton swab. The invading cells were then fixed in 100% methanol and stained with 0.1% crystal violet solution. They were then counted using a microscope (magnification, ×200; (Carl Zeiss MicroImaging, Jena, Germany).

### Quantitative polymerase chain reaction (qPCR)

Total cellular RNA was isolated using the Illustra triplePrep extraction kit in accordance with the manufacturer’s instructions. The quantity of RNA was determined spectrophotometrically. The RNA was then reverse transcribed using the High Capacity RNA-to-cDNA Master Mix (Applied Biosystems™, Foster City, CA, USA). First-strand cDNA was synthesized from 2 μg of total RNA. The PCR primer sequences used were as follows: E-caderin (502 bp) forward, 5′-CGCATTGCCACATACA-3′ and reverse, 5′-CGTTAGCCTCGTTCTCA-3′; Vimentin (690 bp) forward, 5′-CGCTTCGCCAACTAC AT-3′, and reverse, 5′-AGGGCATCCACTTCACAG-3′; β-actin (179 bp) forward, 5′-ATCGTGCGTGACATTAAGGAGAAG-3′ and reverse, 5′-AGGAAGGAAGGCTGGAAGAGTG-3′. PCR was performed under the following conditions: denaturation at 95°C for 30 sec, annealing at 60°C for 30 sec and extension at 72°C for 45 sec. PCR samples were loaded onto a 1.2% agarose gel containing ethidium bromide. All PCR experiments were performed in triplicate. The housekeeping gene β-actin was used as an internal control.

### Western blotting

For isolation of total proteins, control and treated cells were washed in ice cold PBS, lysed in Radioimmunoprecipitation assay buffer (containing 50 mM Tris-base, 150 mM NaCl, 0.1% SDS, 1% Triton X-100, 0.5% sodium deoxycholate, 1 mM sodium orthovanadate, 10 mM sodium fluoride and 1% protease inhibitor cocktail) and quantified using the Bradford protein assay. The cellular lysates were separated using 10% SDS-PAGE, and transferred onto a nitrocellulose membrane. The membranes were blocked with 5% non-fat milk in TBST and incubated with primary antibodies at 4°C overnight. The membranes were then incubated with 1:2,000 horseradish peroxidase-conjugated secondary antibodies for 2 h. Immunoreactive bands were visualized using an enhanced chemiluminescence kit. Western blot signals were quantitated by densitometric analysis using Total Lab Nonlinear Dynamic Image analysis software (MathWorks, Natick, MA, USA).

### Statistical analysis

Each experiment was performed ≥three times. Data are presented as the mean ± standard deviation and the differences were analyzed using Student’s t-test. P<0.05 was considered to indicate a statistically significant difference.

## Results

### Effect of resveratrol on the growth of PCa cells in vitro

**Figure 1:**
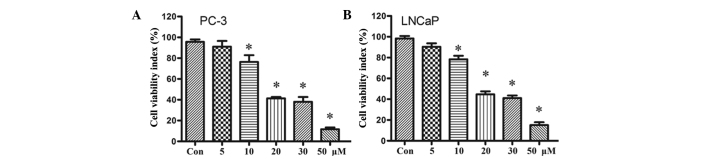
Anti-proliferative effect of resveratrol (0–50 μmol/l) in PC-3 and LNCaP prostate cancer cells. Results are representative of three independent experiments. ^*^P<0.05, vs. untreated group.

The effect of resveratrol on the proliferation of PC-3 and LNCaP cells was investigated. The two cell lines were cultured *in vitro* with different concentrations of resveratrol (0–50 μM) for 48 h, and cell viability was measured using the MTT assay. The results demonstrated that the proliferative abilities of PC-3 and LNCaP cells decreased in the presence of resveratrol in a dose-dependent manner. In addition, the results demonstrated that treatment with resveratrol at concentrations ≤10 μM exhibited no cytotoxic effects on PC-3 and LNCaP cells (Fig. 1). Therefore, lower concentrations of resveratrol, without cytotoxic effects on PC-3 and LNCaP cells, were used for the subsequent experiments.

### Resveratrol inhibits LPS-induced EMT morphological changes in PCa cells

**Figure 2:**
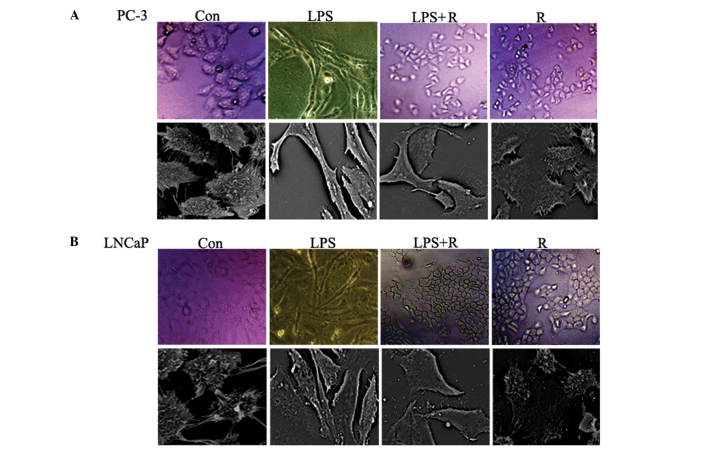
Resveratrol inhibits LPS-induced cell morphological changes characteristic of EMT in PC-3 and LNCaP PCa cells. Cells were incubated with either LPS (5 μg/ml) or LPS plus resveratrol (10 μmol/l). After 48 h, cellular morphological changes were examined using optical (magnification, ×400) and scanning electron microscopy (magnification, ×6,000–10,000). Results are representative of three independent experiments. LPS, lipopolysaccharide; Con, control group; LPS+R: LPS + resveratrol group; R, resveratrol group.

In the present study, it was investigated whether resveratrol may inhibit EMT. LPS-treated PC-3 and LNCaP cell lines were used since LPS (5 μg/ml) has been previously demonstrated to induce EMT (20). Optical and scanning electron microscopy was used to investigate changes in the morphology of PC-3 and LNCaP human PCa cells exposed to LPS, in the presence or absence of resveratrol. Cells were treated with LPS for 48 h. As shown in Fig. 2A and B, the two cell lines underwent typical EMT morphological changes in response to LPS: there was a loss of cell-to-cell contact leaving scattered clusters of cells, the cells acquired a spindle-shaped and fibroblast-like phenotype and scanning electron microscopy revealed that the number of extracellular microvilli increased in certain cells. It was then investigated whether resveratrol was capable of inhibiting these LPS-induced phenomena. The mesenchymal phenotype was less marked in cells co-treated with LPS and resveratrol compared with cells treated with LPS alone (Fig. 2A and B). These results indicate that resveratrol inhibits LPS-induced EMT.

### Resveratrol inhibits the expression of EMT markers in PCa cells

**Figure 3:**
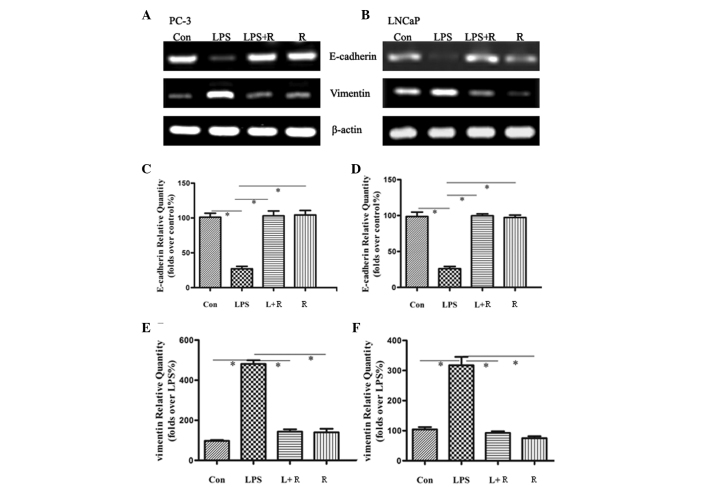
Resveratrol prevents the LPS-induced decrease in E-cadherin mRNA expression and increase in vimentin mRNA expression. (A and B) The mRNA expression levels of E-cadherin and vimentin in (A) PC-3 and (B) LNCaP PCa cells were determined using reverse transcription polymerase chain reaction. (C and D) Quantification of E-cadherin mRNA in (C) PC-3 and (D) LNCaP PCa cells. (E and F) Quantification of vimentin mRNA in (E) PC-3 and (F) LNCaP PCa cells. Data from ≥three independent experiments with duplicate determinations are expressed as the mean ± standard error of the mean. LPS, lipopolysaccharide; Con, control group; LPS+R or (L+R), LPS + resveratrol group; R, resveratrol group. ^*^P<0.05 vs. LPS.

**Figure 4:**
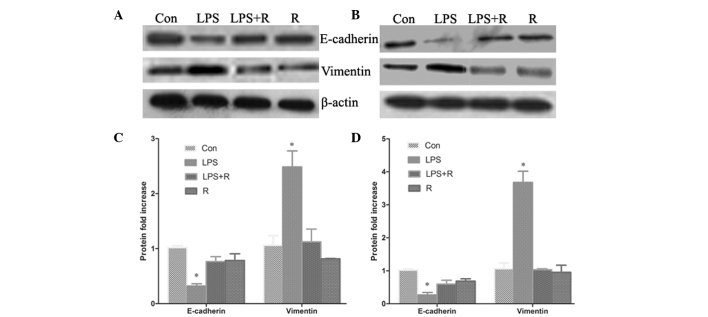
Resveratrol prevents the LPS-induced decrease in E-cadherin protein expression and increase in vimentin protein expression. (A and B) The protein expression levels of E-cadherin and vimentin in (A) PC-3 and (B) LNCaP PCa cells were determined using western blot analysis. (C and D) Quantification of E-cadherin and vimentin protein expression levels. Data from ≥three independent experiments with duplicate determinations are expressed as the mean ± standard error of the mean. LPS, lipopolysaccharide; Con, control group; LPS+R, LPS + resveratrol group; R, resveratrol group. ^*^P<0.05 vs LPS.

In addition to the morphological changes, the expression of EMT phenotypic markers was detected using qPCR and western blot analysis. The results from the qPCR (Fig. 3A–F) demonstrate that the mRNA levels of vimentin and E-cadherin in LPS-treated cells were significantly increased and suppressed, respectively. Western blot analysis (Fig. 4A–D) revealed that the protein expression of E-cadherin was also significantly downregulated in the LPS-treated cells compared with control cells, whilst vimentin protein expression was significantly increased (P<0.05). In cells treated with resveratrol, LPS-induced EMT was found to be reversed, resulting in the induction of E-cadherin expression and the inhibition of vimentin expression (Fig. 3 and 4). These results further suggest that resveratrol has an inhibitory effect on cellular EMT.

**Figure 5:**
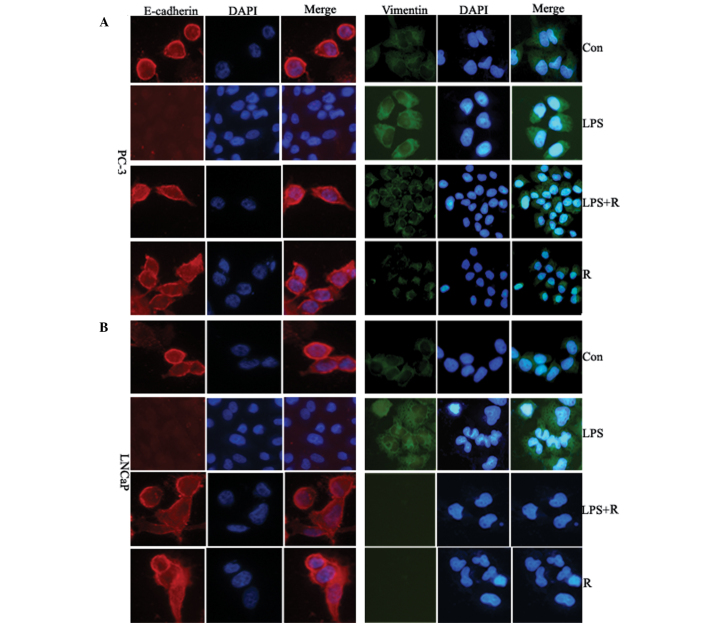
Immunodetection of E-cadherin and vimentin proteins in (A) PC-3 and (B) LNCaP PCa cells. PC-3 and LNCaP cells were incubated with LPS, resveratrol (10 μM) or LPS and resveratrol. After 48 h, fluorescent images were obtained using a confocal laser scanning microscope. The E-cadherin fluorescence signal in the resveratrol group is greater compared with the control group; whilst vimentin is lower compared with the control group. LPS, lipopolysaccharide; Con, control group; LPS+R, LPS + resveratrol group; R, resveratrol group.

To further determine possible alterations in E-cadherin and vimentin, PC-3 and LNCaP cells treated with resveratrol were stained with fluorescence immunostaining and analyzed using confocal microscopy. The E-cadherin fluorescence signal in the resveratrol treated-cells was higher after 48 h compared with untreated cells, whilst the vimentin fluorescence signal was substantially lower (Fig. 5). These results further suggest that resveratrol has inhibitory effect on cellular EMT.

### Hh signaling is required to decrease E-cadherin and increase vimentin expression levels

**Figure 6:**
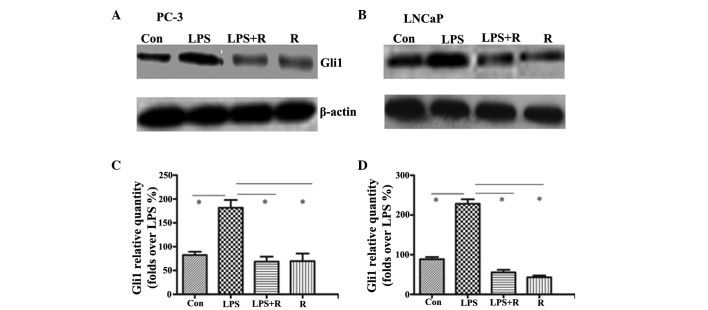
Hedgehog signaling activation is inhibited by resveratrol. (A and B) Western blot analysis of the transcription factor Gli1 in (A) PC-3 and (B) LNCaP cells following treatment with LPS, LPS + R and R. The β-actin protein was used as an internal control. (C and D) Densiometric measurement of Gli1 protein level was normalized against the internal control and expressed as a relative value. ^*^P<0.05 vs. LPS. LPS, lipopolysaccharide; Con, control group; LPS+R, LPS + resveratrol group; R, resveratrol group.

Previous studies have suggested that the Hh signaling pathway may induce EMT of cancer cells. To investigate whether the inhibitory effect of resveratrol is associated with the inhibition of Hh signaling activation, the expression of Gli1 transcription factor was measured in PCa cells using western blot analysis. The results demonstrated that LPS promotes the expression of Gli1 protein, accompanied by a decrease in E-cadherin expression and an increase in vimentin expression. It was found that resveratrol inhibits this effect (Fig. 6). These results indicate that Hh signaling has an important role in the EMT process.

### Resveratrol inhibits invasion of PCa cells

**Figure 7:**
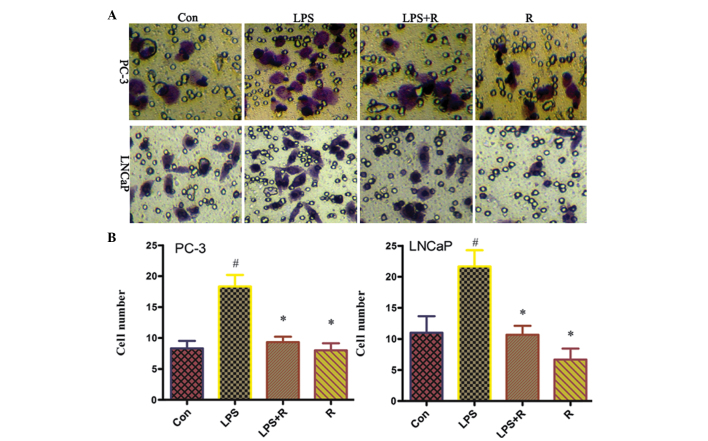
Cell invasion assay in PC-3 and LNCaP cells. LPS significantly stimulated cell invasion, which was inhibited by resveratrol. (A) The bottom side of the filter inserts show cells that have migrated through the filter pores. (B) The number of cells in each treatment group. LPS, lipopolysaccharide; Con, control group; LPS+R, LPS + resveratrol group; R, resveratrol group. ^#^P<0.05, LPS vs. control; ^*^P<0.05, LPS vs. LPS+R, R.

EMT is associated with enhanced cellular progression. To investigate whether resveratrol may inhibit tumor invasion, an *in vitro* invasion assay, using a Matrigel model, was performed. As shown in Fig. 7, following treatment with LPS, the number of invasive cells increased significantly compared with untreated cells. However, the number of invasive cells was significantly reduced in cells co-treated with LPS and resveratrol. These results suggest that resveratrol blocks the ability of LPS to increase the invasiveness of human PCa cells.

## Discussion

EMT has an important role in embryonic development (21); however, it has also recently been implicated in tumor invasiveness (22). The acquisition of EMT phenotype allows cells to metastasize in distant sites, therefore enhancing tumor progression (4).

Previous studies have suggested that LPS may have a role in mediating EMT. The results from the present study demonstrated that PC-3 and LNCaP cells treated with LPS exhibit a spindle-shaped, fibroblastic morphology and express EMT markers. However, LPS-induced EMT in PCa cells (PC-3 and LNCaP) treated with resveratrol was no longer observed. Resveratrol was found to restore the epithelial phenotype in mesenchymal cells and inhibit the expression of LPS-induced EMT markers. It was also shown that resveratrol upregulates the expression of E-cadherin, whilst downregulating the expression of vimentin. In addition, resveratrol inhibits the expression of LPS-induced Gli1 protein. Gli1 is a transcription factor in the Hh signaling pathway, therefore suggesting that it mediates the expression of E-cadherin and vimentin (23). It is likely that the Hh signaling may be involved in LPS-induced EMT in PC-3 and LNCaP PCa cells. These results extend the current understanding of the mechanism by which resveratrol may act to inhibit cancer cell invasiveness.

Resveratrol is a polyphenolic phytoalexin found in grapes and other fruits. Currently, numerous preclinical studies have found that resveratrol is a potential therapeutic agent for cancer prevention and/or treatment. Resveratrol has been shown to retard the growth of various cancer cells through multiple cellular signaling pathways, including the Src-STAT3, NF-κB, Wnt and Hh signaling pathways (17–19,24). Vergara *et al* (25) demonstrated that resveratrol inhibits the epidermal growth factor-induced EMT in MCF-7 cells (25). Li *et al* (26) also reported that resveratrol inhibits EMT in pancreatic cancer cells via suppression of the PI-3K/Akt/NF-κB pathway, and Wang *et al* (27) demonstrated that resveratrol inhibits TGF-β1-induced EMT and suppresses lung cancer invasion and metastasis (27). Furthermore, Chen *et al* (28) reported that resveratrol inhibits LPS-induced EMT in a mouse melanoma model. In the present study, it was found that resveratrol inhibits the expression of Gli1 and downregulates or upregulates the expression of the EMT markers E-cadherin and vimentin, respectively. Additionally, resveratrol inhibits cancer cell invasion, and these results are in accordance with the results from Chen *et al* (28). Furthermore, in the present study, it was demonstrated for the first time, to the best of our knowledge, that the anti-metastatic effects of resveratrol are associated with EMT in PCa cells. These results provide a novel perspective on the role of resveratrol in preventing the progression of cancer.

Hh proteins were first identified in *Drosophilia melanogaster*, and were found to regulate embryonic cell growth and carcinogenesis in certain vertebrate tissues (23). As a signaling pathway, Hh firstly binds to Patched (Ptch), leading to the activation of Gli1 transcription factors and an upregulation of Gli1 target genes (29). The Gli1 transcription factor is an important mediator in the Hedgehog pathway that regulates genes essential for tumor progression (30). Xu *et al* (31) previously demonstrated that sonic hedgehog-Gli1 signals promote EMT by mediating a complex signaling network in pancreatic tumors (31). Gli1 is an important positive regulator of epithelial differentiation and decreased levels of Gli1 are likely to contribute to the highly metastatic phenotype observed in pancreatic ductal adenocarcinoma. In the present study, the effect of resveratrol on Gli1 protein expression, was investigated. It was found that the expression of Gli1 was inhibited, accompanied by an increase in expression of E-cadherin and a decrease in expression of vimentin. Therefore, this suggests that Hh signaling may regulate EMT. However, other mechanisms of regulation that may explain the ability of resveratrol to suppress the EMT process cannot be ruled out. Multiple proteins are targeted by resveratrol, therefore, high-throughput methods may be used in the future. In addition, although it was demonstrated in the present study that resveratrol inhibits EMT, further studies are required to fully elucidate the regulatory mechanism *in vivo*.

Taken together, the results from the present study suggest that the ability of resveratrol to inhibit tumor invasion is associated with the EMT, possibly by inhibiting the activation of the Hh signaling and regulating the expression of the important downstream EMT markers, E-cadherin and vimentin. These results provide a novel mechanistic base for the therapeutic application of resveratrol in patients with PCa.
